# PARP inhibitors combined with ionizing radiation induce different effects in melanoma cells and healthy fibroblasts

**DOI:** 10.1186/s12885-020-07190-9

**Published:** 2020-08-18

**Authors:** Verena Weigert, Tina Jost, Markus Hecht, Ilka Knippertz, Lucie Heinzerling, Rainer Fietkau, Luitpold V. Distel

**Affiliations:** 1 Department of Radiation Oncology, University Hospital Erlangen, Strahlenklinik, Universitätsstraße 27, 91054 Erlangen, Germany; 2 Department of Immune Modulation, University Hospital Erlangen, Hartmannstr. 14, 91052 Erlangen, Germany; 3 Department of Dermatology, University Hospital Erlangen, Hautkrebszentrum, Hautklinik, Ulmenweg 18, 91054 Erlangen, Germany

**Keywords:** Kinase inhibitor, Ionizing radiation, PARP1/PARP2, Cell death, Cell cycle, Homologous recombination, Radiosensitivity

## Abstract

**Background:**

PARP inhibitors niraparib and talazoparib are FDA approved for special cases of breast cancer. PARP is an interesting repair protein which is frequently affected in cancer cells. We studied the combined action of talazoparib or niraparib with ionizing radiation in melanoma cells and healthy fibroblasts.

**Methods:**

Homologous recombination (HR) status in six different melanoma cell lines and healthy fibroblasts was assessed. Cell cultures were treated with PARP inhibitors talazoparib or niraparib and ionizing radiation (IR). Apoptosis, necrosis and cell cycle distribution was analyzed via flow cytometry. Cell migration was studied by scratch assays.

**Results:**

Studied melanoma cell cultures are HR deficient. Studied healthy fibroblasts are HR proficient. Talazoparib and niraparib have congruent effects within the same cell cultures. In all cell cultures, combined treatment increases cell death and G2/M arrest compared to IR. Combined treatment in melanoma cells distinctly increases G2/M arrest. Healthy fibroblasts are less affected by G2/M arrest. Treatment predominantly decelerates or does not modify migration. In two cell cultures migration is enhanced under the inhibitors.

**Conclusions:**

Although the two PARP inhibitors talazoparib and niraparib appear to be suitable for a combination treatment with ionizing radiation in our in vitro studies, a combination treatment cannot generally be recommended. There are clear interindividual differences in the effect of the inhibitors on different melanoma cells. Therefore, the effect on the cancer cells should be studied prior to a combination therapy. Since melanoma cells increase more strongly than fibroblasts in G2/M arrest, the fractional application of combined treatment should be further investigated.

## Background

Kinases play a critical role in cellular signaling. Many of them are associated with human cancer initiation and progression. Therefore, small molecule kinase inhibitors were developed for kinase-targeted cancer therapy. Since the early 1980s, 37 kinase inhibitors (KI) have received FDA approval for treatment of malignancies [1]. Among them are kinase inhibitors targeting key DNA repair proteins such as Poly-ADP-ribose-polymerases (PARPs). Already striving for genomic instability, cancer cells preferably use less accurate DNA repair named non-homologous end joining (NHEJ) [2]. The predominant lack of genetic stability severed by PARP inhibition could therapeutically be exploited by adding radiotherapy. Radiotherapy inactivates cancer cells mainly by inducing DNA damage. Kinase inhibitors can act as radiosensitizer, when simultaneously applied with ionizing radiation. Exemplarily, in vitro and in vivo studies demonstrated that PARP inhibitor LT626 in combination with ionizing radiation acted synergistically inhibiting growth in lung and pancreatic cancers [3].

It is also known, that patients with genetic instability and impaired DNA repair ability can have drastically increased reactions after radiotherapy [4]. Patients, who react more distinctively to irradiation and therefore show significant side effects, are possibly radiosensitive. This is based on genetic differences like short-nucleotide-polymorphism (SNP), mutations in caretaker proteins or DNA-damage-repair related proteins like ataxia telangiectasia mutated (ATM) [5]. In those cases, enhanced radiosensitivity is associated with serious side effects. When V600E-mutation-specific BRaf-inhibitor vemurafenib was compared to dabrafenib, it induced radiosensitivity to a much higher degree and thus provoked side effects [6, 7]. When stereotactic body radiotherapy is practiced with concurrent BRAF inhibitors, it is advised to pause inhibitors at least 1 week before radiotherapy [8]. Further information about the interaction of kinase inhibitors and irradiation is needed, in order to assess whether a simultaneous treatment should be advised to optimize cancer treatment. In this context, toxicity to healthy tissue and efficacy to eliminate cancer tissue should be considered.

**Fig. 1:**
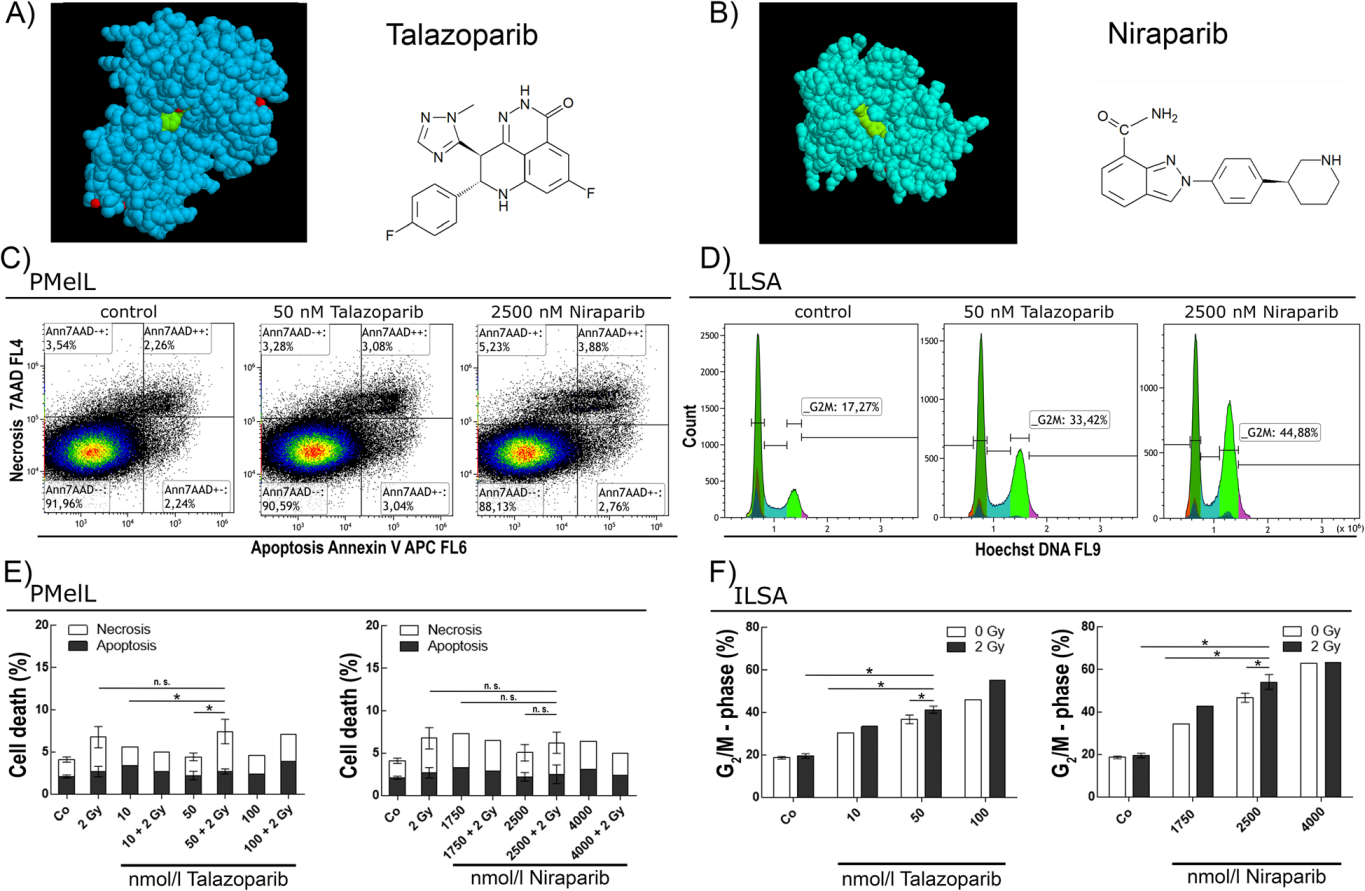
Talazoparib and niraparib in combination with irradiation induces apoptosis and necrosis and cell cycle arrest. **a** Left: talazoparib (blue) bound in PARP1 [12], right: structural chemical formula of talazoparib. **b** Left: niraparib (green) bound in PARP1 [13], right: structural chemical formula of niraparib. **c** Exemplary gating strategy of Annexin-V-APC/7AAD staining for flow cytometry detection for apoptosis and necrosis. Dot plots of melanoma cell culture PMelL untreated, treated with 50 nmol/l talazoparib or 2500 nmol/l niraparib. **d** Representative histograms of Hoechst stained DNA distribution in melanoma cell culture ILSA untreated, treated with 50 nmol/l talazoparib or 2500 nmol/l niraparib. **e** Left: dose escalation study of apoptotic and necrotic PMelL cells treated with 0 nmol/l up to 100 nmol/l talazoparib w/o 2 Gy IR. right: dose escalation study of apoptotic and necrotic PMelL cells treated with 0 nmol/l up to 4000 nmol/l niraparib w/o 2 Gy IR **f** Left: dose escalation study of G2/M phase in ILSA cells treated with 0 nmol/l up to 100 nmol/l talazoparib w/o 2 Gy IR. Right: dose escalation study of G2/M phase in ILSA cells treated with 0 nmol/l up to 4000 nmol/l niraparib w/o 2 Gy IR. Bars without error bars have one repetition (*n* = 1) and bars with error bars have three or four repetitions (*n* = 3 or *n* = 4), * = *p* ≤ 0.05

In 2017, the PARP inhibitor niraparib (ZEJULA, Tesaro Inc., Waltham, USA) (Fig. 1b) was approved for maintenance therapy of recurrent platinum sensitive ovarian, fallopian tube or primary peritoneal cancer by the FDA [9]. One year later, the PARP inhibitor talazoparib (TALZENNA, Pfizer Inc.) (Fig. 1a) was approved for adult patients with deleterious or suspected deleterious gBRCAm, HER2-negative, locally advanced or metastatic breast cancer by the FDA [10]. In advanced or metastatic situations radiotherapy is commonly used to treat cancer patient [11].

As both PARP inhibitors are small molecule NAD^+^ mimetics, they are designed to block the catalytic activity of PARP1 and PARP2 and therefore impair DNA repair (Fig. 1a, b) [14]. Talazoparib is a more potent but less selective PARP1/2 inhibitor (PARP1 IC50 = 0.57 nmol/l) [15]. Niraparib also is a more selective inhibitor for PARP1 (IC50 = 3.8 nmol/l) and PARP2 (IC50 = 2.1 nmol/l) [16, 17]. Their mechanism of PARP trapping interferes with single strand break repair and provokes DNA double strand breaks by collapse of stalled replication forks. Furthermore, PARP inhibitors evoke synthetic lethality in homologous recombination (HR) deficient cells [18, 19].

We were moreover interested in the interaction between PARP inhibitors niraparib and talazoparib and ionizing radiation, since just few data is available for this combination. Both KI were approved for patients with advanced or metastatic situations, where cell migration plays a crucial role. We examined, whether the combined treatment of PARP inhibitors and ionizing radiation alters the therapeutic effect in different melanoma and healthy tissue cell cultures.

## Methods

### Cell culture and inhibitor

SBLF7 and SBLF9 are human skin fibroblasts extracted from healthy donors. Therefore, the primary human fibroblasts SBLF7 and SBLF9 were isolated from a healthy donor by skin biopsy and subsequently cultivated [20]. BIMA, ILSA and RERO are human melanoma cells that originate from primary tumors of patients diseased with melanoma [21]. Primary human melanoma cells (from primary tumors) were collected in the Department of Dermatology of the University Hospital of Erlangen following approval by the institutional review board and cultured 50 passages maximum. Therefore, we defined these cells as cell cultures in contrast to cell lines. Single cell suspensions were generated by digesting tissue samples with collagenase (Sigma, Darmstadt, Germany), hyaluronidase (Sigma, Darmstadt, Germany), and DNAse (Roche, Mannheim, Germany) [22]. A375M, PMelL and Mel624 are purchasable melanoma cell lines provided by the Department of Immune Modulation at the University Hospital of Erlangen. Fibroblasts were cultured in F-12 (Gibco, Waltham, USA), supplemented with 15% FBS (Merck, Darmstadt, Germany), 2% NEA (Merck, Darmstadt, Germany) and 1% penicillin/streptomycin (Gibco, Waltham, USA). Melanoma cell cultures were cultured in RPMI-1640 (Sigma Aldrich, München, Germany), supplemented with 20% FBS (Merck, Darmstadt, Germany), 1% NEA (Merck, Darmstadt, Germany), 1% Pyruvate-solution (Gibco, Waltham, USA), 1% L-Glutamine (Merck, Darmstadt, Germany), 1% HEPES (Merck, Darmstadt, Germany), and 0.05% Gentamicin (Merck, Darmstadt, Germany). All cells were incubated at 37 °C in a humidified 5% CO_2_ atmosphere.

Niraparib (Selleck Chemicals LLC, Huston, USA) and talazoparib (Selleck Chemicals LLC, Huston, USA) diluted in DMSO were stored at − 80 °C. Required aliquots were freshly thawed prior to each experiment. Prior to usage, talazoparib was thinned with PBS (Sigma Aldrich, St. Louis, USA) at a ratio of 1:100. This dilution of the stock solution enabled adequate pipetting for the required amounts of talazoparib.

### Immunostaining based homologous recombination assay

Cells were cultured on cover slips to 90% confluence maximum. Medium was exchanged and one-half of cells was treated with 5 μmol/l of a DNA-PK inhibitor (CC-115) for 24 h. Cells were irradiated with 10 Gy and fixed with 4% formaldehyde after 4 h. Slides were blocked with BSA overnight and stained with γH2AX (1:1500, Merck, Darmstadt, Germany) and RAD51 (1:250, abcam, Cambridge, UK) primary antibodies overnight at 8 °C. Secondary antibodies AlexaFluor488 goat anti-mouse (1:400) and AlexaFLuor594 chicken anti-rabbit (1:200) were used (Invitrogen, Eugene, USA). Cover slips were transferred onto glass slides using Vectashield (Vector Laboratories, Burlingame, Canada). For detection, a Zeiss Axio Plan 2 fluorescence microscope at 400x magnification (Zeiss, Oberkochen, Germany) was used. In minimum 150 cells per sample were analyzed and calculated separately. The automated analysis was performed by BIOMAS software (MSAB, Erlangen, Germany), which was especially trained for counting foci in DAPI-stained cell nuclei.

### Apoptosis and necrosis analysis by flow cytometry

Cells were seeded in an appropriate concentration to reach a confluence of 50% up to 80% in 24 h up to 72 h within their cell culture flasks. After 24 h, the incubation medium was changed to a serum-reduced cell culture medium (2% FBS) with different drug concentrations. Concerning talazoparib, cells were treated with an amount of none, 10 nmol/l, 50 nmol/l and 100 nmol/l of inhibitor in each cell culture flask. For niraparib, cells were treated with the amount of none, 1750 nmol/l, 2500 nmol/l and 4000 nmol/l of inhibitor in each cell culture flask. Reduced cell culture medium was used to not artificially enhance the effect of inhibitor. After 3 h, half of the cell culture flasks were irradiated with 2 Gy ionizing radiation (IR) by ISOVOLT Titan X-ray generator (GE, Ahrensburg, Germany). After another 48 h of incubation at 37 °C, cells including the supernatant were harvested. Cells were stained with Annexin V-APC (BD, Heidelberg, Germany) and 7-amino-actinomycin D (7-AAD) (BD, Heidelberg, Germany) for 30 min on ice to analyze apoptosis and necrosis via the Cytoflex flow cytometer (Cytoflex, Beckman Coulter, Brea, USA) [23]. Kaluza Analysis software (Beckman Coulter, Krefeld, Germany) was applied for data evaluation. Cells showing low staining (Annexin-neg./7-AAD-neg.) were defined as “viable” cells, whereas Annexin-pos./7-AAD-neg. Cell were defined as “apoptotic” and double positive cells (Annexin-pos./7-AAD-pos.) as “necrotic” populations (e.g. in Fig. 1c, d).

### Cell cycle by flow cytometry

Seeding and treatment procedure was identical to apoptosis and necrosis analysis. After harvesting, the cells were fixed in 10 ml of 70% ethanol (Roth, Karlsruhe, Germany) and 1 ml of serum-reduced cell culture medium (2% FBS) for a minimum of 12 h at 4 °C. Then, the cells were stained with Hoechst 33258 (Invitrogen, Eugene, USA) for 60 min on ice. The distribution over the different cell cycle phases was analyzed using flow cytometry and Kaluza Analysis software.

### Cell migration assay

The cell migration assay was used to describe the migration behavior of melanoma cells and fibroblasts. Cells were seeded and incubated for 24 h at 37 °C, generating monolayers in the cavities of cell culture plates. To provoke starvation, the incubation medium was replaced by serum-reduced cell culture medium (2% FBS). After another 24 h, the monolayer in each cavity was scratched with a 10 μl pipet tip, creating precise cell-free wounds. Concerning talazoparib, cells were treated with an amount of none or 50 nmol/l of inhibitor in serum-reduced cell culture medium. For niraparib, cells were treated with the amount of none or 2500 nmol/l of inhibitor in serum-reduced cell culture medium. After 3 h, half of the cell culture plates were irradiated with 2 Gy. To record migration, images of the same scratch area were repeatedly taken by a microscope at 100x magnification (Zeiss Primo Vert, Oberkochen, Germany) over 48 h of treatment (Fig. 4a – d). The varying area of the cell wound over time was measured by an image analyzing software (Biomas, MSAB, Erlangen, Germany). A smoothing algorithm filtered the images and set the area at point 0 h to 100%, proportionally calculating the following scratch areas. The changing relative scratch area over the time of 48 h was then outlined in graphs (Fig. 4e – f). For tangible comparison of the individual conditions differing in their treatment, the area under each curve was evaluated [24]. The area under the curve (AUC) was calculated in between the time points 0, 24 and 48 h that resulted in two measurable areas between 0 h and 24 h, and 24 and 48 h. The mathematically based maximum value of one area is “1”. Therefore, the highest achievable AUC value is “2”, when no migration is detectable.

### Statistics

Graphs were generated using scientific software GraphPad Prism 8 (GraphPad Software, San Diego, USA). The data of cell death and cell cycle flow cytometry was analyzed by the unpaired, one-tailed Mann–Whitney U–test. The cell migration assays were evaluated by the unpaired, two-tailed Mann–Whitney U–test.

## Results

We studied PARP inhibitors talazoparib (Fig. 1a) and niraparib (Fig. 1b) in combination with ionizing radiation. We used three patient-derived melanoma cell cultures, three melanoma cell lines and two healthy-donor-fibroblast cell cultures derived from normal skin biopsies.

In the beginning of our study, we initially looked at cell death and cell cycle distribution.

Regarding cell death, the dot plots for PMelL (Fig. 1c) show exemplarily how the flow cytometry data were evaluated. Cells treated with kinase inhibitor were gated in populations representing apoptotic, necrotic and viable cells. The histograms for ILSA (Fig. 1d) visualize the gating strategy of flow cytometry data concerning cell cycle distribution. Each cell line underwent a dose escalation study for cell death and cell cycle distribution (Fig. 1e, f).

The mean (± SD) peak plasma concentration published by the FDA approval “PRESCRIBING INFORMATION” (c_max_) of talazoparib (16.4 ng/ml) and niraparib (800 ng/ml) in vivo served as reference point for applied concentrations of inhibitor which were approximately 50 nmol/l for talazoparib and 2500 nmol/l for niraparib [25, 26].

### Homologous recombination assay

**Fig. 2:**
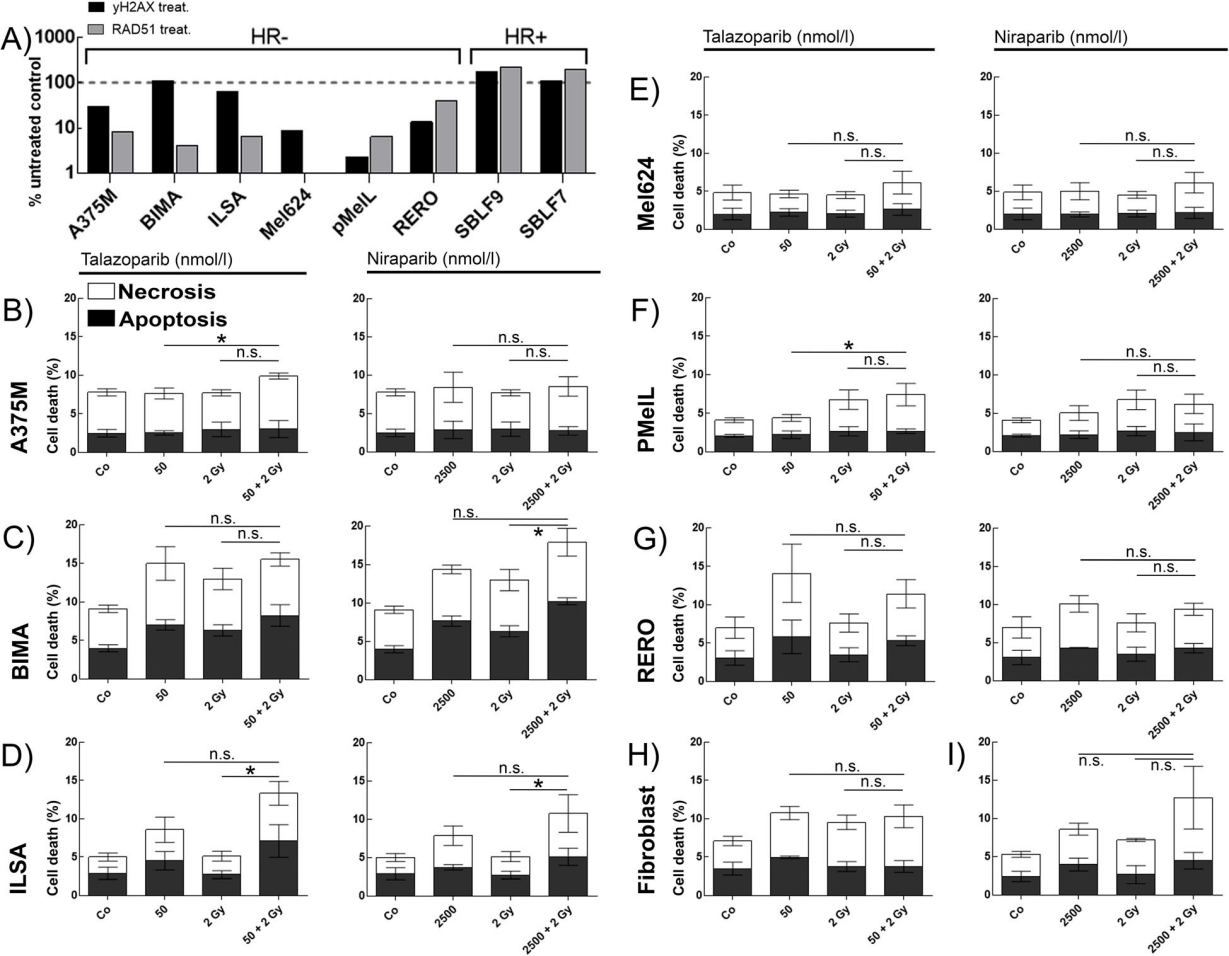
Homologous recombination status and cell death in melanoma and healthy tissue cells. **a** Homologous recombination activity evaluated by DNA DSB induction and repair 24 h after exposure to DNA-PK inhibitor CC-115 and 10 Gy irradiation. Decreased remaining γH2AX foci and RAD51 foci indicates deficiency in homologous recombination. Apoptotic and necrotic rates in melanoma cells **b** A375M, **c** BIMA, **d** ILSA, **e** Mel624, **f** PMelL, **g** RERO and healthy donor skin fibroblasts **h** SBLF9, **i** SBLF7 treated w/o 50 nmol/l talazoparib, w/o 2 Gy IR or w/o 2500 nmol/l niraparib, w/o 2 Gy IR; * = *p* ≤ 0.05; each value represents mean ± SD (*n* = 3 or *n* = 4)

Foci of γH2AX and RAD51 in minimum 150 analyzed cell were counted to analyze the activity of homologous recombination (HR). γH2AX identifies the irradiation-induced DNA double strand breaks. RAD51 is a protein of the signal cascade of the HR. We treated the cells with a DNA-PK inhibitor and forced them to utilize HR. HR-proficient cells should be able to use HR for DNA repair and the numbers of RAD51 foci should be increased. In contrast, HR-deficient cells should have stable or decreasing numbers of RAD51 foci. We found that healthy donor fibroblasts increase their count of RAD51 foci and are therefore HR-proficient. After DNA-PK inhibition RAD51 foci decrease in all malignant cell cultures and cell lines. They are therefore defined as HR-deficient (Fig. 2a).

### Apoptosis and necrosis

Following our dose escalation study of each cell culture, we then chose to present the most relevant treatment concentrations in detail. Therefore, cells were treated with the mean (± SD) peak plasma concentration (c_max_) in vivo of 50 nmol/l talazoparib or 2500 nmol/l niraparib, IR alone and the combination of inhibitor and IR. Induction of apoptosis and necrosis was analyzed by Annexin V-APC and 7-AAD staining and measured via flow cytometry (Fig. 2).

Overall talazoparib and niraparib provoke a similar response pattern regarding cell death within melanoma cells and healthy fibroblasts. Regarding the comparison of IR and combination therapy IR + KI, treatment related effects allow us to group the seven tested cell cultures into non-responder, moderate-responder and strong-responder.

Looking at the inhibitor talazoparib, fibroblasts (Fig. 2h) and PMelL (Fig. 2f) do not seem to respond to combination therapy (IR + talazoparib) compared to IR (non-responder). A375M (Fig. 2b), BIMA (Fig. 2c), Mel624 (Fig. 2e) and RERO (Fig. 2g) are identified as having a moderate response to the combination of KI and IR (moderate-responder). Finally, only ILSA (Fig. 2d) shows a significant increase in combination therapy (*p* = 0.0286) and can therefore be defined as a strong-responder for talazoparib compared to all other tested cell cultures.

The second inhibitor niraparib presents almost the same response pattern in all seven tested cell cultures. Looking at the treatment responses more deeply sheds light on a slight change in grouping the cell cultures. A375M (Fig. 2b), PMelL (Fig. 2f) and RERO (Fig. 2g) do not display more or less cell death using IR + KI (non-responder). Mel624 (Fig. 2e) and the healthy fibroblasts (Fig. 2i) show slight tendencies to increase cell death moderately (moderate-responder). BIMA (Fig. 2c) and ILSA (Fig. 2d) respond significantly (BIMA, *p* = 0.05); ILSA, *p* = 0.0286) with more cell death after combination of niraparib with irradiation.

### G2/M arrest in cell cycle

**Fig. 3:**
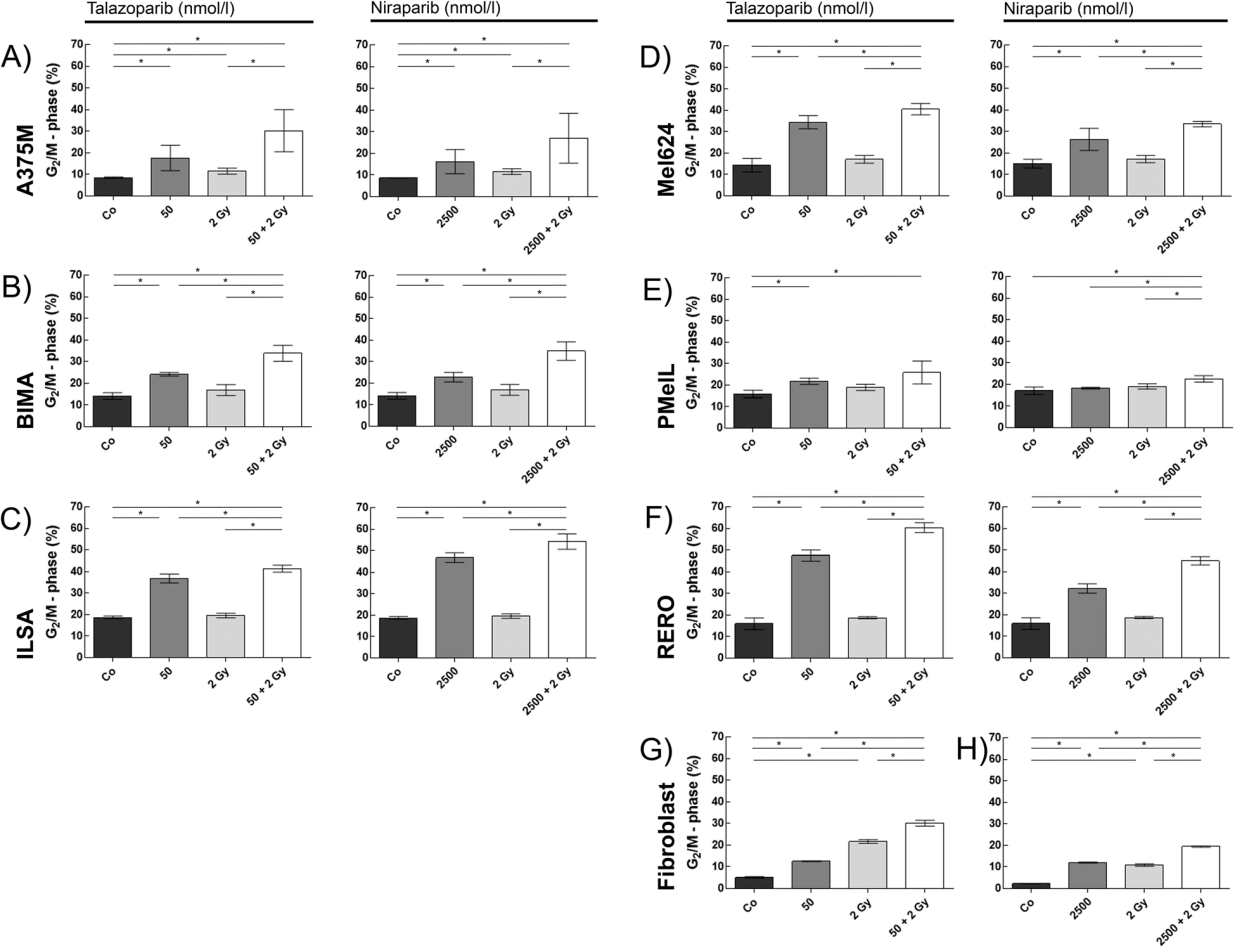
Distribution of G2/M phase in melanoma and healthy tissue cells. The amount of G2/M phase was analyzed in melanoma cells **a** A375M, **b** BIMA, **c** ILSA, **d** Mel624, **e** PMelL, **f** RERO and healthy donor skin fibroblasts **g** SBLF9, **h** SBLF7 treated w/o 50 nmol/l talazoparib, w/o 2 Gy IR or w/o 2500 nmol/l niraparib, w/o 2 Gy IR; * = *p* ≤ 0.05; each value represents mean ± SD (*n* = 3 or *n* = 4)

Cell cycle distribution was analyzed by Hoechst 33258 staining and flow cytometry. The amount of G2/M phase arrest was quantified 48 h after treatment (Fig. 3).

Both PARP inhibitors generate the same tendencies of G2/M arrest within melanoma cells.

In all cell cultures (except PMelL + niraparib), a significant increase of the G2/M population after inhibitor treatment can be seen, when compared to the untreated control group. Fibroblasts, representing healthy tissue, and melanoma cell cultures, representing tumor tissue, behave congruently.

To address the question of radiosensitivity in our cell cultures, the control groups were compared to the irradiated groups (co vs. 2 Gy). Only healthy fibroblasts and A375M increase significantly in their amount of G2/M phase arrest (*p* = 0.05). All other cell cultures are not influenced by IR alone.

The most relevant comparison for our radiation oncology context is the effect of IR alone and of IR combined with KI treatment. In all cell cultures a clear increase of G2/M phase can be seen, when combined treatment (KI + IR) is compared to IR (A375M, BIMA, Mel624, PMelL and fibroblasts *p* = 0.050; ILSA and RERO *p* = 0.028). This finding is telling for our clinical work. Patients with relapse, occurring metastases or inoperable tumors are irradiated commonly. Even cases of disease under kinase inhibitor treatment can progress and an additional irradiation is gaining importance. To improve the treatment for these patient cases we want to clarify if a sequential or simultaneous treatment during irradiation is beneficial in a cell biology setting.

### Cell migration

**Fig. 4:**
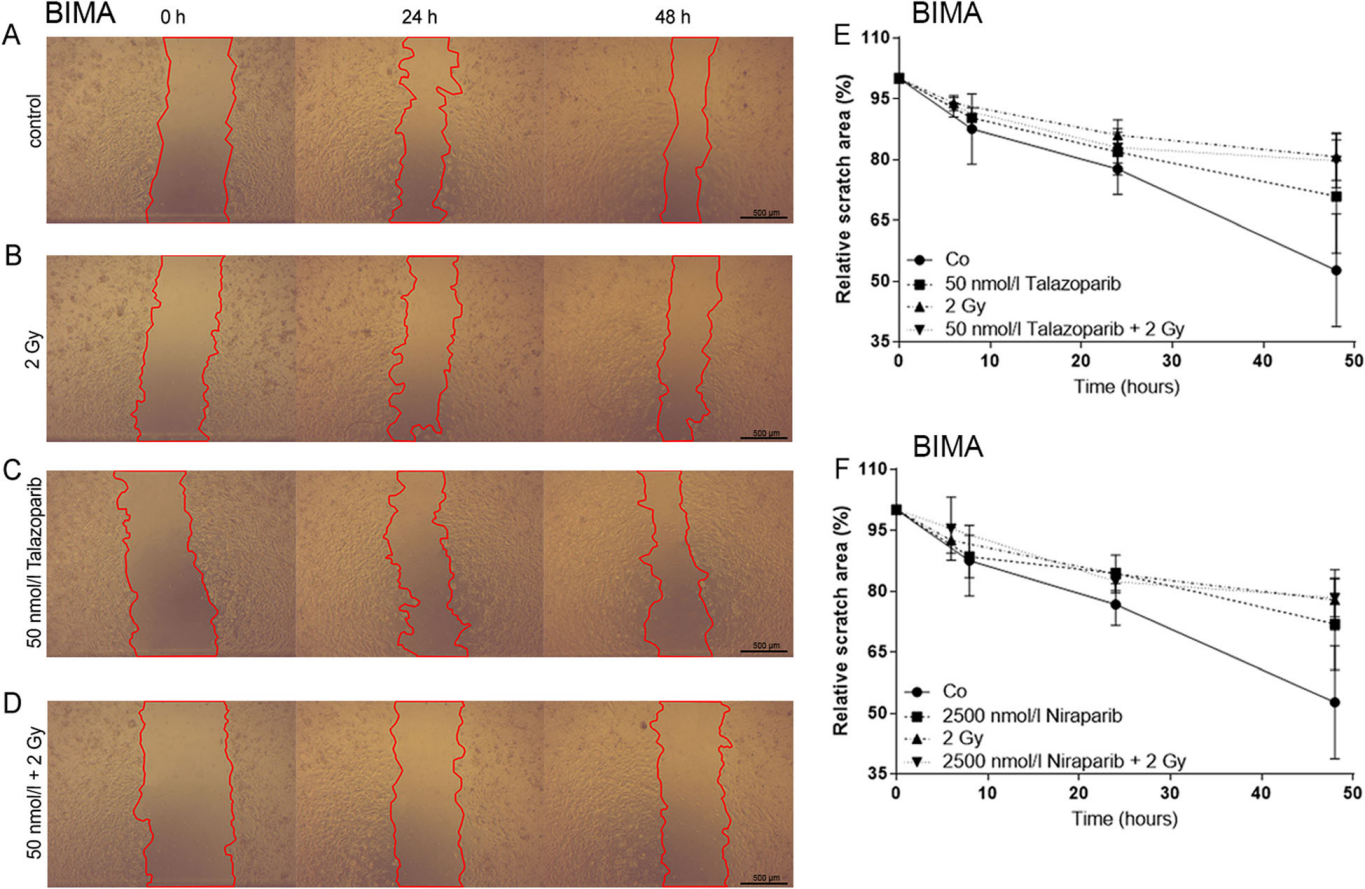
Cell migration analysis in melanoma cells. **a** Representative scratch area of melanoma cell line BIMA without treatment **a**, treated with 2 Gy IR **b**, treated with 50 nmol/l talazoparib **c** and with combined treatment of 50 nmol/l talazoparib and 2 Gy IR **d** over time (0 h, 24 h, 48 h). **e** Decrease of the relative scratch area of melanoma cell line BIMA w/o talazoparib treatment, w/o 2 Gy IR within 48 h; each value represents mean ± SD (*n* = 4). **f** Decrease of the relative scratch area of melanoma cell line BIMA w/o niraparib treatment, w/o 2 Gy IR within 48 h; each value represents mean ± SD (*n* = 4)

**Fig. 5:**
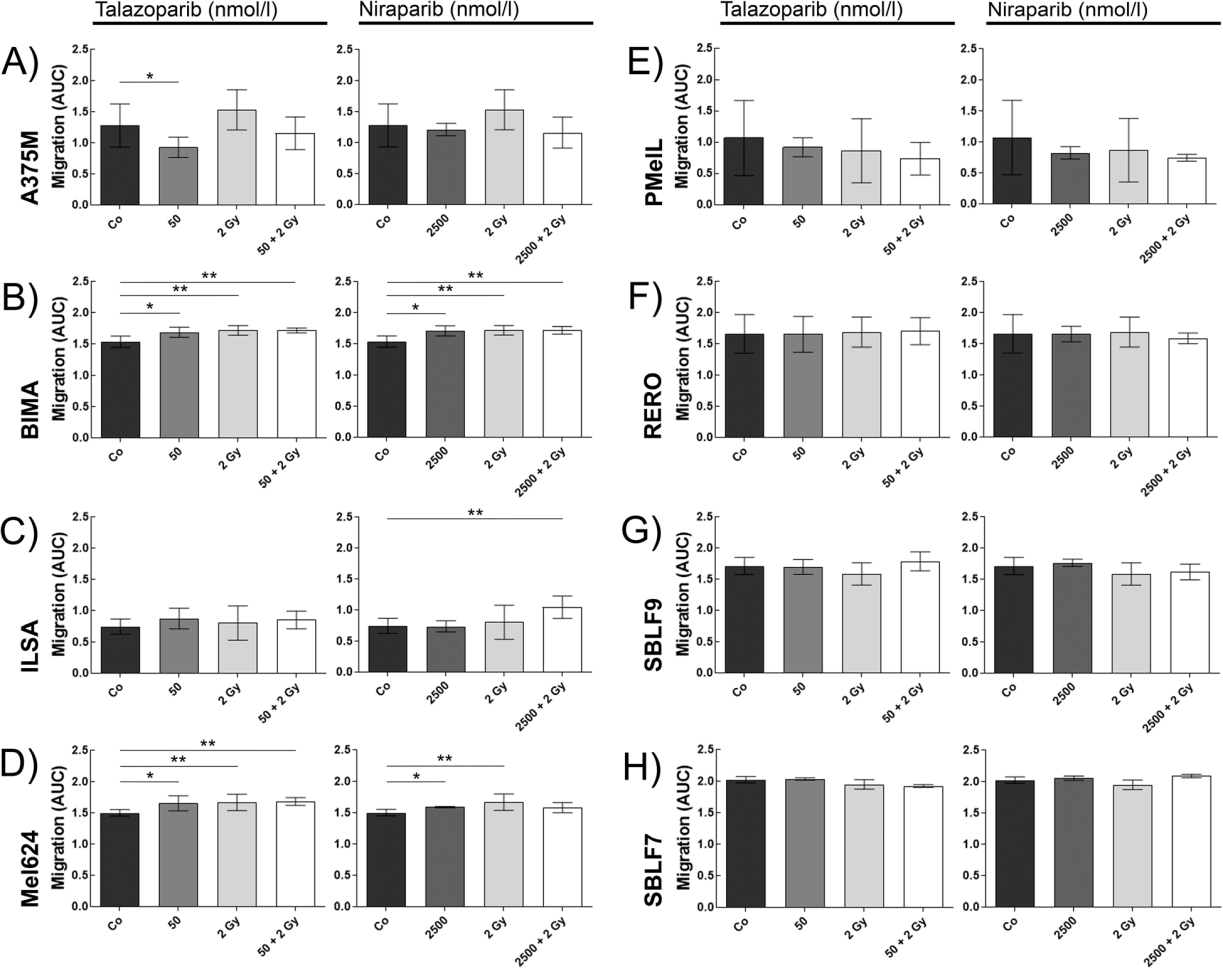
Area under the curve describing migration behavior in melanoma and healthy tissue cells. Melanoma cells **a** A375M (*n* = 4), **b** BIMA (*n* = 4), **c** ILSA (*n* = 4), **d** Mel624 (*n* = 4), **e** PMelL (*n* = 4), **f** RERO (*n* = 4) and healthy tissue cell cultures **g** SBLF9 (*n* = 5), **h** SBLF7 (*n* = 5) treated w/o 50 nmol/l talazoparib, w/o 2 Gy IR or w/o 2500 nmol/l niraparib, w/o 2 Gy IR; the area under the curve of the relative scratch area within 48 h describing migration behavior (AUC = 2 = no migration, AUC < 2 = migration); * = *p* < 0.05, ** = *p* < 0.01, *** = *p* < 0.001; each value represents mean ± SD

Additionally, the influence of the inhibitors talazoparib and niraparib on cell migration was studied. Therefore, cell-free wounds were scratched into monolayers of starved cells. Images of the same scratch area were acquired over 48 h (Fig. 4a – d) and the cell free area was computed. Both inhibitors have an equivalent influence on the same cell culture (Fig. 4e, 5f). Cells that underwent treatment were studied in their amount of migration compared to the control group. Migration under any treatment is decreased in BIMA, ILSA and Mel624 (Fig. 5b - d). Within these cell cultures, migration decreases in combined treatment (BIMA: talazoparib + IR *p* = 0.009, niraparib + IR *p* = 0.009; ILSA: niraparib + IR *p* = 0.008; Mel624: talazoparib + IR *p* = 0.004). The drug treatment tends to enhance migration in PMelL (Fig. 5e). Migration in cell cultures RERO, SBLF7, SBLF9 (Fig. 5f – h) is not changed by treatment. Only the inhibitors alone (50 nmol/l talazoparib: *p* = 0.048) or inhibitors in combination with IR increase migration in melanoma cell line A375M (Fig. 5a). Irradiation alone lessens cell migration in this cell line compared to its control group.

## Discussion

PARP inhibitors block single strand break repair and lead to an increased amount and complexity of DNA damage. Normally, homologous recombination deals with the resulting DNA lesions. Lacking HR, melanoma cells do not pass the G2/M checkpoint. Hereby, the cells are kept from undergoing mitotic division, meaning the rate of proliferation in these malignant melanoma cells is considerably reduced [27, 28]. In all studied melanoma cell cultures, the HR activity was impaired (deficient) while healthy tissue fibroblasts were HR proficient. Notably, former studies showed that treatment with niraparib did lead to a significant longer progression-free survival independent from HR status in patients [29]. Both inhibitors induce comparable effects, but niraparib requires a 50 times higher concentration. Skin biopsies were taken from different melanoma patients and the biopsies were cultivated in cell culture until cells migrated out of the tissue. Previous BRaf-inhibitor-treatment assays showed that ILSA, BIMA and RERO respond to vemurafenib treatment suggesting a BRaf-V600E-mutation. Further characterization of our cell cultures using a neoplasm-specific mutation-panel should be done.

In summary, it is seen that the six tested melanoma cell cultures behave quite diversly when treated with IR and KI. Amongst other things, this could originate from the primary character of BIMA, RERO and ILSA, all generated from skin biopsies in the department of dermatology of the Universitätsklinikum Erlangen. Additionally, no difference between malignant and non-malignant cells could be presented here.

The overall effects of combining talazoparib or niraparib with irradiation can be better observed when analyzing additive or supra-additive effects. Therefore, we calculated whether the combination therapy causes more cell death than the individual effects of 2 Gy and inhibitor treatment. Talazoparib combined with irradiation leads to a decrease of cell death in fibroblasts, BIMA and RERO. The combination shows more effect than the added up values of single treatment. This suggests a radioprotective impact. A375M, Mel624 and ILSA increase their cell death rate in a supra-additive manner, which could root in a radiosensitization triggered by the inhibitor. PMelL showed the same amount of cell death.

Niraparib leads to a slightly different pattern of response. A375M and BIMA outline no greater synergy between IR and KI. PMelL and RERO show less cell death under combination treatment than the effects of the single treatment combined. This gives a hint at a radioprotective effect. Finally, the healthy fibroblasts, Mel624 and ILSA present an increase of cell death by the combination therapy compared to the calculated value of both single treatments. This suggests a radiosensitizing effect in these cell cultures.

In both, healthy fibroblasts and melanoma cells, the combined treatment compared to irradiation treatment increases cell death and G2/M arrest. Thereby, the increase in G2/M arrest is more distinct than the increase in cell death under combined treatment compared to IR.

A radiosensitizing effect of both drugs has been reported before. Talazoparib had radiosensitizing effects in small cell lung cancer cell lines in short-term viability and clonogenic survival assays [30]. Clonogenic survival analyzes indicated enhanced radiosensitivity in tumor cell lines derived from lung, breast and prostate cancers [31]. Newest findings of Wang et al. 2020 showed a radiosensitizing effect of niraparib in head and neck squamous cancer cells too [32].

The most sensitive cell cycle phases to IR are G2 and mitosis [33]. G2/M-phase arrest increases by a factor of 1.8 to 3.1 in all melanoma cell cultures with the exception of PMelL when radiotherapy is compared with combined treatment and by a factor of 1.1 to 1.7 when chemotherapy is compared with combined treatment. In contrast, the amount of G2/M phase in fibroblasts only increases within the range of 1.4 up to 1.7 when radiotherapy is compared to combined treatment. Thus, G2/M phase is increased to a lesser extent in healthy fibroblasts by combined treatment compared to melanoma cells. An increasing G2/M phase in melanoma cells prior to radiotherapy may enhance the efficiency of radiotherapy. A growing synchronization of cells in these most radiosensitive phases of cell cycle could be achieved by simultaneously applying the inhibitor to a fractionated radiation treatment [33]. Hence, healthy tissue could be preserved by this approach in treatment [34].

We additionally focused our analysis on calculating additive and supra-additive (synergistic) effects. Our findings suggest that combining IR with KI treatment leads to higher impact on increasing G2/M than adding the single effects (KI) + (IR) in five out of seven tested cell cultures (except PMelL and fibroblasts). This supports our thesis of enhancing the effect of irradiation by simultaneously adding DNA-damage-repair-inhibitors.

Furthermore, we wanted to assess the influence of the inhibitors on cell migration. Inhibitor treatment does not influence migration in RERO, SBLF7and SBLF9. It decelerates migration in BIMA, ILSA and Mel624. The tendency of the combined treatment reducing cell migration makes this approach in treatment acceptable and even favorable regarding metastatic tendencies in these cell cultures. In contrast, A375M and PMelL enhance cell migration after inhibitor treatment with and without IR. Since both cell lines did not differ from the other tested malignant cell lines in HR-status (deficient) and neither cell death nor cell cycle analysis hinted at a special behavior of A375M and PMelL, we were not able to find an explanation for the unexpected migration behavior. Scratch assays examine a more collective way of migration. An additional migration assay like a transwell assay would be recommended for further investigation. Our clinical context forces us to focus on the question if patients should be irradiated sequentially or simultaneously to a kinase inhibitor treatment. Considering that, no changes are detectable when combining PARP inhibitors to irradiation treatment, leads us to the assumption that migration is not influence by talazoparib or niraparib based on our data presented here.

In summary, cell death increases in melanoma cells after treatment with PARP inhibitor and irradiation (Fig. 4) which could be the effect of a rising G2/M arrest induced by PARP inhibitors. Regarding the increase of G2/M phase arrest, we could prove a significant increase when inhibitor was combined with IR. Similarly, healthy fibroblasts were affected by the combined treatment. The overall comparison of talazoparib (more potent, less selective) and niraparib (more selective) influencing the effect of irradiation on a cellular level results in a similar outcome within the same cell line. Concerning clinical radiation therapy, it is to be further investigated if fractionated application of combined treatment could enhance harm to tumor tissue while sparing healthy tissue. In general, our data suggest that PARP inhibitors niraparib and talazoparib lead to enhanced toxic effects in combination with irradiation. However, this outcome can only be very carefully related to a clinical application. Nevertheless other research groups found an increased effect in healthy tissue of C57BL6 mice, too [35].

The limitations of the work are that it is in vitro experiments that can only partially predict the effect in humans. However, they do give an indication from the multiple melanoma cell cultures how different the effect might be in different individuals. A proof of mechanisms causing these effects would support the theses and must be provided in further work.

## Conclusion

The two PARP inhibitors talazoparib and niraparib are suitable for combined treatment with ionizing radiation from the perspective of in vitro studies. Nevertheless, it seems that combinatorial treatment cannot be generally recommended. There are clear interindividual differences in the effect of the inhibitors on different melanoma cells. Therefore, the effect on cancer cells should be studied prior to a combination therapy. Nevertheless, combinatory treatment could serve as an option to get treatment results, when tumor tissue appears resistant to radiation.

## Data Availability

The datasets used and analyzed during the current study are available from the corresponding author Prof. Dr. Luitpold Distel on reasonable request.

## Abbreviations

7-AAD: 7-amino-actinomycin D
APC: Allophycocyanin
ATM: Ataxia Telangiectasia Mutated
AUC: Area under the curve
DMSO: Dimethyl sulfoxide
DNA: Deoxyribonucleic acid
DNA – PK: DNA – dependent protein kinase
DSB: Double strand break
FACS: Fluorescence associated cell sorting
FBS: Fetal bovine serum
FDA: Food and drug administration
Gy: Gray
HR: Homologous recombination
IR: Ionizing radiation
KI: Kinase inhibitor
NEA: Non-essential amino acid
NHEJ: Non-homologous end joining
PARP1: Poly-ADP-ribose-polymerase 1
PARP2: Poly-ADP-ribose-polymerase 2
PBS: Phosphate-buffered saline
SD: Standard deviation
SNP: Short-nucleotide-polymorphism
